# Combined intracardiac echocardiography and pressure-sensing catheter for complex PFO closure: a case report

**DOI:** 10.3389/fcvm.2025.1623207

**Published:** 2025-07-23

**Authors:** Rui Yan, Xiaofang Li, Jun Li, Xin Shao, Anxin Zhang, Liping Guo, Fanqi Li, Haixiong Wang

**Affiliations:** ^1^ Department of Cardiology, Shanxi Cardiovascular Hospital, Taiyuan, Shanxi, China; ^2^ Department of Digestive Oncology, Cancer Center, Shanxi Bethune Hospital, Shanxi Academy of Medical Sciences, Tongji Shanxi Hospital, Third Hospital of Shanxi Medical University, Taiyuan, Shanxi, China; ^3^ Department of Cardiology, The Second Xiangya Hospital of Central South University, Changsha, China

**Keywords:** no.18, Yifen street, Taiyuan city, Shanxi province, China no.99, Longchengda street, China

## Abstract

**Background:**

While transcatheter closure of a patent foramen ovale (PFO) is usually technically feasible, certain anatomical subtypes such as long-tunnel PFOs can present considerable challenges. Due to the difficulty in advancing the guidewire through the elongated and tortuous tunnel, interventional cardiologists often have to resort to transseptal puncture. However, transseptal puncture is associated with a higher incidence of postoperative residual shunts and an increased risk of puncture-related complications. In this case report, we describe for the first time a novel approach that combines intracardiac echocardiography (ICE) with a pressure-sensing catheter to facilitate guidewire advancement during the PFO procedure. We refer to this innovative technique as the “push and easy” method. This approach offers a promising solution for the closure of complex long-tunnel PFOs while minimizing radiation exposure during the procedure.

**Case summary:**

A 20-year-old male patient with a complex long-tunnel PFO was referred to our center. Initial attempts to visualize and traverse the PFO using ICE alone were unsuccessful due to the tunnel's excessive length and tortuosity. Consequently, a pressure-sensing catheter was introduced. This catheter, offering real-time pressure feedback along with enhanced support and maneuverability, enabled successful guidewire passage through the PFO. Under the combined guidance of ICE and the pressure-sensing catheter, precise and safe closure of the complex PFO was achieved, without any radiation exposure. We refer to this novel, radiation-free approach as the “push and easy” technique, which may offer a valuable option for the management of complex long-tunnel PFOs.

## Introduction

PFO is a common anatomical condition and a potential contributor to paradoxical embolism (1). Currently, transcatheter closure is the most widely adopted treatment technique (2). However, this procedure can be technically challenging in cases with a “long-tunnel” PFO morphology. In such cases, transseptal puncture is often regarded as a last-resort option when standard techniques prove unsuccessful. Moreover, this approach carries a higher risk of postoperative residual shunting and puncture-related complications, and thus is typically reserved for highly selected patients (3).

With the advancement of ICE, detailed visualization of intracardiac structures has become possible, and ICE has been reported to assist in the closure of long-tunnel PFOs (4, 5). However, in particularly complex cases, ICE guidance alone may be insufficient to ensure successful passage. Radiofrequency ablation catheters equipped with pressure-sensing capabilities offer a potential advantage in such scenarios, as they can detect real-time pressure changes at the atrial septum and provide superior support and steerability compared to conventional guidewires.

To our knowledge, the application of pressure-sensing catheters in PFO closure procedures has not been previously reported. Herein, we present the first documented case of a complex long-tunnel PFO successfully closed under the combined guidance of ICE and a pressure-sensing catheter.

## Case presentation

**Figure 1:**
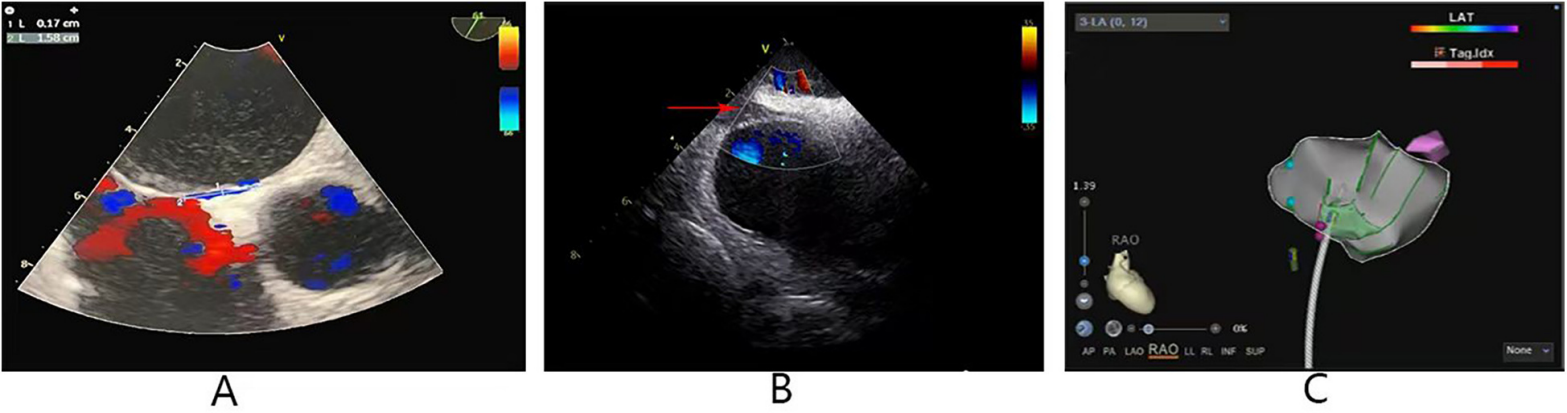
**(A)** Transesophageal echocardiogram showing a PFO: length 15.8 mm, width 1.7 mm. **(B)** The red arrow indicates that ICE view of PFO. **(C)** The Pink dots indicate PFO locations. PFO, patent foramen ovale; ICE, intracardiac echocardiography.

A 20-year-old male patient was referred to our hospital with a 6-month history of recurrent headaches following a failed attempt at PFO closure at another institution. From the previous cardiac intervention procedure report, the PFO was occluded using the traditional fluoroscopic guidance. Unfortunately, the guidewire was unable to pass through the PFO after repeated attempts, and the occlusion failed. Transthoracic echocardiography revealed a left-to-right atrial-level shunt, and confirmed a long-tunnel–type PFO measuring approximately 1.7 mm in width and 15.8 mm in length (Figure 1A). The patient's Risk of Paradoxical Embolism score was 10, suggesting a high probability that the PFO was causally related to the clinical symptoms. Given the prior unsuccessful intervention, a novel approach was adopted to achieve successful closure.

The procedure was performed as follows: After local anesthesia, the right femoral vein was punctured, and 8F and 11F sheaths were inserted. Using a Soundstar ICE catheter (Biosense Webster, Irvine, CA), 11F in diameter, the morphology of the PFO can be clearly visualized and the PFO location can be marked within the ICE. (Figures 1B,C; Supplementary Video S1). However, after several attempts, the guidewire still could not pass through the PFO, possibly due to the long and tortuous tunnel of the PFO and the soft tip of the guidewire.

**Figure 2:**
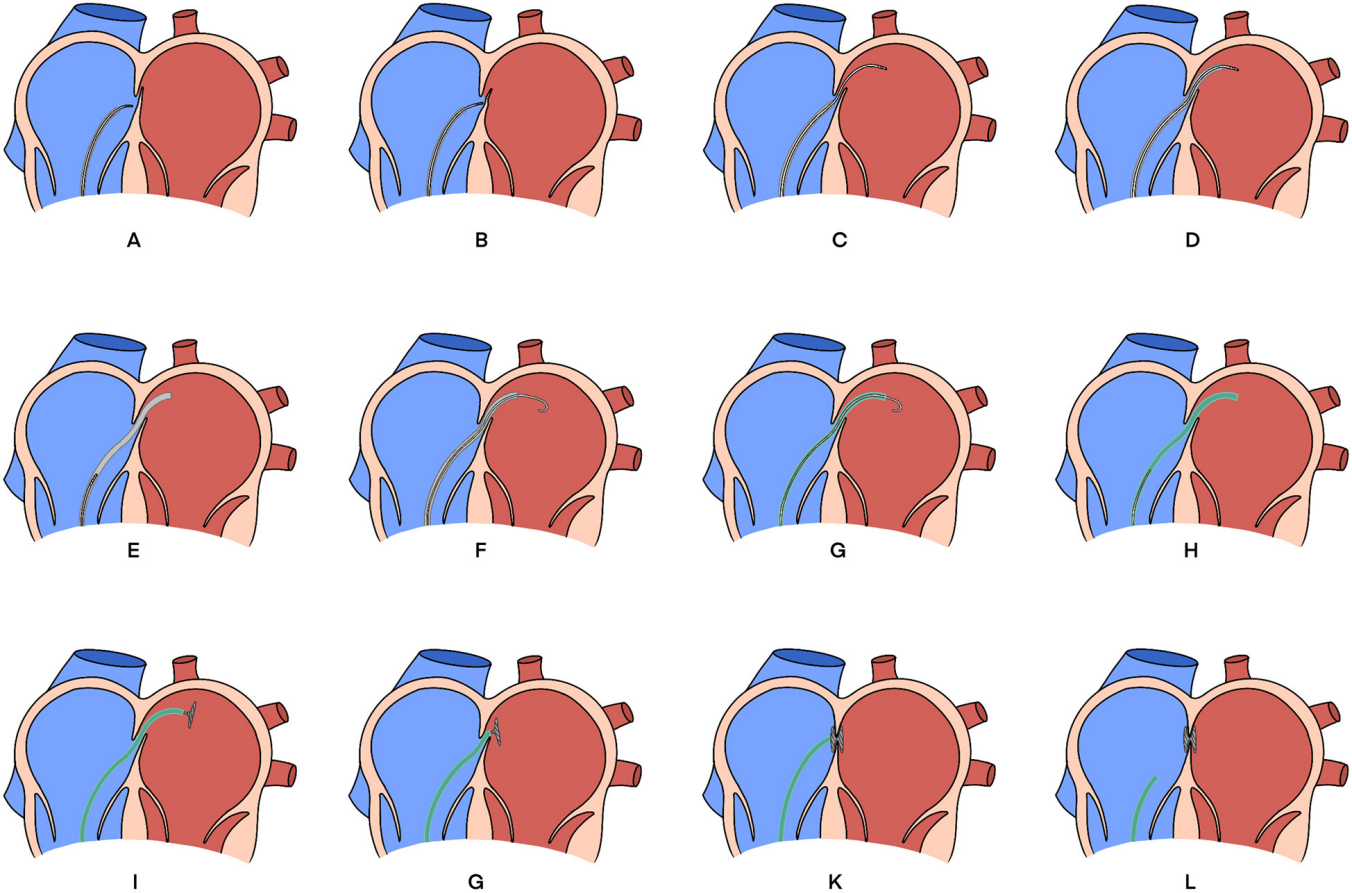
Schematic diagram of the steps for PFO occlusion. **(A)** The SmartTouch catheter was first placed near the PFO. **(B)** Pushed the catheter forward to artificially increase the width of the PFO. **(C)** Pushed the catheter through PFO into LA. **(D)** Delivered the sheath along the catheter into the LA. **(E)** the catheter was removed, and the 8F-sheath was retained in the LA. **(F)** Delivered the guidewire through the sheath into the LA. **(G)** Replaced 8F sheath with 12F sheath using guidewire exchange technique. **(H)** Withdrew the guidewire, and advanced the PFO occluder into the LA. **(I)** Unfolded the LA disk. **(J)** Retracted the delivery system to close the left septum of the PFO. **(K)** Unfolded the right atrial disk. **(L)** Repeated pushing and pulling to confirm the occlusion stability, and then released the occluder. PFO, patent foramen ovale; LA, left atrium.

Subsequently, a SmartTouch catheter (Biosense Webster, Irvine, CA) was introduced into the right atrium via an 8F sheath (Fast-CathTM, Abbott). The pressure-sensing feature of the ablation catheter can sense the pressure on the contact surface in real time, and the vector arrows at the tip of the head end can show the current direction of the catheter in real time. Similar to the procedure for radiofrequency ablation of atrial fibrillation, we used an ICE catheter to reconstruct the structures of the right and left atria. Under real-time ICE guidance, the catheter was positioned near the PFO. With the aid of the catheter's contact force sensing function, the SmartTouch catheter was advanced under 3D guidance to push open the PFO. When the tip pressure decreased, the catheter successfully traversed the PFO (Supplementary Video S2). The catheter was then advanced into the left superior pulmonary vein (LSPV), and the 8F sheath was advanced until its tip entered the left atrium (Supplementary Video S3). After withdrawing the catheter from the 8F sheath, a long guidewire was advanced through the 8F sheath into the LSPV. The 8F sheath was then removed, and a 12F sheath (Shanghai Shape Memory) was advanced through the PFO to the middle of the left atrium along the guidewire. A PFO occluder (1825, Shanghai Shape Memory) mounted on the delivery rod was then advanced to the middle of the left atrium along the sheath. Retracting the sheath allowed the left atrial disc to unfold naturally. The delivery system was then retracted to approximate the left septum of the PFO (Supplementary Video S4), and finally, the right atrial disc was unfolded. Repeated pushing and pulling confirmed the stability of the occluder, and ICE was used to verify the proper positioning of the occluder. Finally, the occluder was successfully released (Supplementary Video S5). The entire procedure took 77 min with zero radiation exposure. The schematic diagram of the entire process is shown in Figures 2A–L. We refer to this approach as the “push and easy” technique. Postoperative antiplatelet and anticoagulant therapy was administered.

A six-month postoperative follow-up revealed no abnormalities of the occluder, no residual shunt, and no right-to-left shunt on contrast echocardiography of the right heart. The patient's migraine symptoms were completely alleviated.

## Discussion

The foramen ovale is a physiological channel in the interatrial septum during embryonic development of the heart. In most individuals, it undergoes anatomical closure within the first year after birth. Persistence beyond the age of three is referred to as a PFO, with a prevalence estimated at 20%–34% in the general population (1). Numerous studies have confirmed that PFO is associated with ischemic stroke and migraine with aura (6). Several large-scale randomized controlled trials have demonstrated that transcatheter closure of a PFO significantly reduces the risk of recurrent stroke and is superior to medical therapy alone in secondary stroke prevention (7–9). Moreover, transcatheter closure of a PFO offers multiple advantages, including minimal trauma, rapid recovery, reliable efficacy, procedural simplicity, low risk of complications, and compatibility with future cardiac surgeries (10, 11). These features make it a safe and effective therapeutic option, especially for high-risk patients who are not suitable candidates for open-heart surgery.

Although transcatheter closure of a PFO is an effective therapeutic approach, it remains technically challenging in certain anatomical subtypes—particularly in cases of long tunnel-type PFOs. A long tunnel-type PFO is typically defined as having a length of ≥8 mm (12); in the case we reported, the patient's PFO measured 15.8 mm in length (Figure 1A). For long tunnel-type PFO, where the guidewire often fails to pass through the long and tortuous tunnel, transseptal puncture is often resorted to as a relatively conventional alternative (13). However, transseptal puncture leads to a higher incidence of postoperative residual shunts and an increased risk of puncture complications (3). Therefore, this technique may be the last option for patients with complex PFOs. With the development of ICE technology, intracardiac structures have become more visible. Studies have demonstrated that ICE is an excellent guide in PFO occlusion procedures, as it facilitates a detailed investigation of the device implantation site (4, 5).

Currently, ICE is widely used in the interventional therapy of arrhythmia and structural heart disease. The use of ICE can greatly reduce the amount of radiation exposure of medical staff, while its real-time monitoring function can improve the success rate and safety of the procedure (14, 15), especially in some special populations (16). However, despite the advantages of high-resolution imaging, dynamic imaging, interference-free imaging, and zero radiation, there is a certain learning curve for ICE catheterization, and the operator needs to accumulate experience to master the technical essentials. Moreover, compared with TEE, there are certain limitations in the imaging field of view and angle, and there is a lower 3D temporal and spatial resolution, and at the same time, the high price of ICE has become a major challenge for its restricted use.

Therefore, in complex PFOs, it is challenging to succeed with a standard PFO closure technology guided solely by ICE. To address these challenges, additional guidance techniques may be necessary. Under the guidance of a three-dimensional mapping system, a steerable pressure-sensing radiofrequency ablation catheter offers dual advantages: it can detect electrical potentials at the fossa ovalis and sense real-time pressure changes. A sudden drop in pressure at the catheter tip indicates passage through the PFO, which not only enhances the success rate of crossing but also ensures procedural safety. However, the use of such pressure-sensing catheters in PFO procedures has not been previously reported. Given its strong support, excellent maneuverability, and real-time pressure feedback, we utilized this catheter in a patient with a complex long tunnel-type PFO. Ultimately, with the combined assistance of ICE and the pressure-sensing catheter, successful closure of the complex PFO was achieved.

The current technique for PFO occlusion is to pass the guidewire through the PFO radiographically, with or without ICE to finalize the occlusion process, and the key step of this technique is whether the guidewire can pass through the PFO smoothly. Although fluoroless occlusion of the PFO has become a reality with the help of ICE, it is difficult to pass the guidewire through the conventional method described above due to the lack of support at the tip of the guidewire in this long tunnel-type complex PFO, whereas the pressure-sensing catheter, with its combination of strong support and pressure-sensing, can be easily and safely pushed away from the PFO to enter the left atrium. At the same time, such a technique can be easily generalized to operators who are skilled in the use of ICE and pressure catheters.

## Limitation

The limitations of this technique include a steep learning curve for both ICE and the use of pressure ablation catheters, as these technologies are complex and require significant time and experience to master. Additionally, the cost is higher compared to traditional occlusion methods.

## Conclusion

The combination of ICE and a pressure-sensing catheter represents a promising strategy for the effective closure of complex long-tunnel PFOs.

## Data Availability

The raw data supporting the conclusions of this article will be made available by the authors, without undue reservation.
